# Supplementary material for: Chaetomium globosum from Alisma orientale (Sam.) Juzep. enhances the antioxidative stress capacity of Caenorhabditis elegans
Source: PeerJ. 2025 Aug 13;13:e19827. doi: 10.7717/peerj.19827 (PMC12357546; doi:10.7717/peerj.19827)
Supplement: Supplemental Information 2 [file peerj-13-19827-s002.docx]

Supplementary Material

***Chaetomium globosum* from *Alisma orientale* (Sam.) Juzep. enhances the antioxidative stress capacity of *Caenorhabditis elegans***

**Nayu Shen^1†^, Zhao Chen^2^****^†^, Siyu Wang^1^, Mingqi Zhang^1^, Yujie Jia^1^, Xinyu Zhang^1^, Yirong Xiao^3^, Zizhong Tang^1^, Qingfeng Li^1^, Ming Yuan^1^, Tongliang Bu^1^**

^1^College of Life Sciences, Sichuan Agricultural University, Ya’an, China

^2^Ya’an People’s Hospital, Ya’an, China

^3^ Sichuan Agricultural University Hospital, Ya’an, China

**^†^**These authors share first authorship

Correspondence Author:

Zizhong Tang^1^

Xinkang Road, Ya’an, Sichuan, 625000, China

Email address: [67031988@qq.com](mailto:67031988@qq.com)

Supplementary Table 1 Effect of CGE on the viability of *C. elegans*

| Concentration (μg/mL) | Survival tate(%) | P-value |
| --- | --- | --- |
| Control | 98±1 | - |
| 10 | 98±1 | >0.05 |
| 40 | 98±1 | >0.05 |
| 70 | 98±1 | >0.05 |
| 100 | 99±1 | >0.05 |
| 150 | 98±2 | >0.05 |
| 200 | 98±1 | >0.05 |

Supplementary Table 2 Statistical analysis of survival time of *C. elegans*

| Stressors | Group | Mean time^(1)^ | P-Value^(2)^ | Average percentage increase^(3)^ | Median time^(4)^ | P-Value^(2)^ |
| --- | --- | --- | --- | --- | --- | --- |
| Ultraviolet stress | Control | 4.70±0.06_a_ | - | - | 4.13±0.10_a_ | - |
|  | Res^（5）^ | 5.34±0.04_c_ | <0.001 | 13.62% | 4.75±0.05_c_ | <0.001 |
|  | 20 μg/mL | 5.02±0.12_b_ | <0.05 | 6.71% | 4.43±0.13_b_ | <0.05 |
|  | 60 μg/mL | 5.20±0.09_b_ | <0.01 | 10.65% | 4.62±0.10_bc_ | <0.05 |
|  | 100 μg/mL | 5.39±0.18_c_ | <0.001 | 14.78% | 4.79± 0.17_c_ | <0.001 |
| oxidative stress | Control | 2.32±0.11_a_ | - | - | 1.98±0.09_a_ | - |
|  | Res^（5）^ | 3.11±0.30_b_ | <0.01 | 34.12% | 2.69±0.24_b_ | <0.01 |
|  | 20 μg/mL | 2.78±0.03_b_ | <0.05 | 19.72% | 2.38±0.02_b_ | <0.05 |
|  | 60 μg/mL | 2.85±0.13_b_ | <0.05 | 22.85% | 2.49±0.11_b_ | <0.05 |
|  | 100 μg/mL | 3.11±0.30_b_ | <0.01 | 33.84% | 2.73± 0.27_b_ | <0.01 |

Note: All data were expressed as Mean±SD, and different letters in the column indicated statistically significant differences (P<0.05). (1) Mean survival time = 1/n∑jXjdj, where j is the age, dj is the number of nematodes dying at Xj, and n is the total number of nematodes. (2) The CGE treatment group was compared with the control group, and the P-value was calculated by log-rank test. (3) Percentage increase in life expectancy relative to the control group. (4) Median life is the time when the survival rate is equal to 50%. (5) 20 μg/mL resveratrol was a positive control.

Supplementary Table 3 Effect of CGE on malondialdehyde content and antioxidant enzyme activity in *C. elegans*

|  |  | Control | Res | 20（μg/mL） | 60（μg/mL） | 100（μg/mL） |
| --- | --- | --- | --- | --- | --- | --- |
| MDA  (nmol/mgprot) | non-oxidative stress | 0.385±0.027^a^ | 0.267±0.014^b^ | 0.298±0.058^b^ | 0.252±0.016^b^ | 0.234±0.12^b^ |
|  | oxidative stress | 1.432±0.089^c^ | 0.871±0.057^a^ | 0.972±0.008^a^ | 0.965±0.019^a^ | 1.165±0.069^b^ |
| SOD  (U/mgprot) | non-oxidative stress | 6.018±0.256^a^ | 6.866±0.158^b^ | 6.597±0.05^b^ | 6.208±0.093^a^ | 6.084±0.031^a^ |
|  | oxidative stress | 7.022±0.564^a^ | 8.337±0.546^c^ | 7.985±0.419^b^ | 7.124±0.027^a^ | 7.071±0.000^a^ |
| GSH-PX  (U/mgprot) | non-oxidative stress | 1.393±0.179^a^ | 4.012±0.253^b^ | 3.091±0146^b^ | 3.245±0.364^b^ | 2.915±0.960^b^ |
|  | oxidative stress | 2.230±0.729^a^ | 5.809±1.240^b^ | 2.376±0.689^a^ | 4.684±1.224^b^ | 5.537±0.603^b^ |
| CAT  (U/mgprot) | non-oxidative stress | 1.128±0.074^a^ | 1.562±0.152^a^ | 1.541±0.140^a^ | 1.780±0.304^b^ | 1.324±0.292^a^ |
|  | oxidative stress | 1.381±0.465^a^ | 2.828±0.880^b^ | 2.113±0.236^a^ | 2.637±0.139^b^ | 1.854±0.215^a^ |

Note: The absence of the same letter in the same column indicates a statistically significant difference (P < 0.05).
